# Facile synthesis of ultratough conductive gels with swelling and freezing resistance for flexible sensor applications

**DOI:** 10.1038/s41598-025-86541-7

**Published:** 2025-03-01

**Authors:** Pengpeng Lu, Jingyang Xu, Shuyan Liu, Lili Fu, Shengxian Wu, Ze Liu, Tu Hou, He Liu, Dongyan Huang

**Affiliations:** 1 College of Engineering and Technology, Jilin Agricultural University, Changchun, 130118 People’s Republic of China; 2 College of Information Technology, Jilin Agricultural University, Changchun, 130118 People’s Republic of China; 3 Key Laboratory of Bionic Engineering, Ministry of Education, Jilin University, Changchun, 130022 People’s Republic of China

**Keywords:** Gels and hydrogels, Colloids

## Abstract

The application of flexible hydrogel sensors in extreme environments, such as low temperatures, underwater, or significant mechanical deformations, poses considerable challenges. Here, we present a simple one-pot method to fabricate ultra-tough, swelling- and freezing-resistant conductive organohydrogels without external conductive and freeze-resistant fillers. During gelation, by-products (C_6_H_15_NHCl, KCl) provide both conductivity and antifreeze properties, thus eliminating compatibility issues and dispersion challenges associated with external fillers. The resulting gel exhibits super toughness, with tensile strength reaching 10.2 MPa and stretchability up to 800% in the dry state. Following covalent crosslinking, the gel demonstrates excellent anti-swelling properties, with a swelling ratio of only 15.4% after 24 h of water immersion, while maintaining a tensile strength of 5.8 MPa and an elongation of up to 1000%. When fabricated into flexible sensors, these gels display stable electrical responsiveness and desired Gauge Factor (0.58–2.25), effectively detecting limb movements. Furthermore, the gel’s superior resistance to freezing and swelling ensures reliable signal stability under both − 20 °C and underwater conditions. These combined properties render the conductive gel a promising candidate for flexible sensing components in robotic and bionic applications.

**Supplementary Information:**

The online version contains supplementary material available at 10.1038/s41598-025-86541-7.

## Introduction

Flexible sensors have attracted substantial attention from academia and industry due to their broad applications in human motion detection^1^, physiological monitoring^2–4^, or plant growth assessment^5^. These sensors often required to function in complex and demanding environments, which necessitates the use of materials that possess high mechanical flexibility, electrical conductivity, and durability^6^. Consequently, flexible conductive materials, particularly ion-conductive hydrogels, have emerged as promising candidates for flexible electronics, owing to their exceptional mechanical flexibility, biocompatibility, and conductivity^7^. However, most existing hydrogels remain prone to freezing, swelling in low-temperature or underwater environments, which leads to mechanical degradation and signal distortion, thereby limiting their practical applicability. Therefore, the advancement of hydrogels with stable performance under various conditions, including low temperature and underwater, is crucial for enhancing the reliability and effectiveness of flexible sensors^8^.

To improve the adaptability of hydrogel materials in diverse environmental conditions, researchers have developed several strategies. For example, a heterogeneous structure was engineered by incorporating “soft” hydrophobic microregions, facilitated by inter- and intramolecular hydrogen bonding and π-π stacking, alongside “hard” crosslinking via strong ionic ligand interactions. This combination resulted in a gel with enhanced resistance to solubility and increased durability, making it particularly well-suited for flexible sensing applications^9^. Additionally, high-performance nanofillers, including carbon black (CB), carbon nanotubes (CNTs)^10^, graphene oxide (GO)^11^, polyaniline (PANI)^12^, and MXenes^13,14^, were physically or chemically integrated with polymer networks to create conductive nanocomposite hydrogels. These nanocomposites effectively balance mechanical strength and electrical conductivity, facilitating the development of durable and stable hydrogel-based sensor devices^15^. Researchers have targeted the freezing of water at low temperatures can obstruct ion channels and reduce flexibility^16,17^. One strategy to prevent freezing in hydrogels involves utilizing hydrogen bonding to secure water molecules within the gel. By incorporating solutes such as glycol, glycerol^18,19^ or cationic/anionic groups^20^ in the gel matrix, the disruption of water’s hydrogen-bond network lowers the freezing point of the hydrogel, thereby effectively preventing freezing and maintaining the material’s flexibility. Additionally, polar solvents such as dimethyl sulfoxide (DMSO) are incorporated to create mixed-solution organohydrogel systems, thereby enhancing the gel’s anti-freezing properties and water retention. This enhancement results from the strong hydrogen bonding between DMSO and water molecules^21,22^. The main differences between hydrogels and organohydrogels are the type of solvent and the hydrophilic/hydrophobicity of the material. Hydrogels offer a promising green alternative to conventional polymers due to their nontoxicity, biodegradability, biocompatibility, environmental friendliness^23^. Organohydrogels are formed by dispersing immiscible hydrophilic/hydrophobic polymer networks or simple hydrophilic polymer networks in organic/aqueous solvent systems. It not only inherits the advantages of hydrogels well, but also exhibits some unique properties, such as freezing and water retention ability, and solubility resistance, which makes it a candidate for constructing favorable environmentally tolerant flexible sensors^24^. Hydrogels typically contain a high concentration of hydrophilic components, leading to substantial water absorption and inevitable swelling^25^. Swelling in aqueous environments decreases mechanical strength and limits the applicability of hydrogels in underwater environments, especially in biomedical fields, posing a significant obstacle to practical use^26^. To address this, anti-swelling hydrogels have been developed by carefully adjusting the hydrophilic-lipophilic balance^27,28^, cross-linking density^29^, and monomer composition^30^. Although optimizing molecular structure can help mitigate hydrogel swelling, existing methods frequently rely on complex formulations, which restrict the ability to dynamically control swelling properties.

Polyvinyl alcohol (PVA) is widely used in flexible sensor materials for its flexibility, transparency, and biocompatibility^31^. The tunability of PVA allows it to optimize its mechanical and sensing properties through cross-linking, composites, or blending with other polymers. Furthermore, PVA materials have high processing adaptability and can be adapted to a wide range of flexible substrates to meet the design needs of different flexible sensors. However, it exhibits notable limitations in terms of mechanical stability, anti-freezing and swelling resistance. Various modification strategies, including the incorporation of toughened polymers and ionic liquid antifreeze agents, have been employed to enhance the mechanical properties and freezing resistance of PVA gels^32–34^. Nonetheless, many toughening approaches remain limited, as modifications often either improve deformability at the expense of tensile strength or enhance tensile strength while compromising gel ductility^35^. To mitigate excessive swelling in water, chemical hydrogels typically incorporate dense chemical crosslinking, which embrittle the hydrogels^36^. There is a lack of straightforward methods for producing PVA gels at a large scale that strike a balance between strength and flexibility, reduce the need for conductive and antifreeze additives, and ensure resistance to both freezing and swelling.

In this study, we synthesize low-density, covalently crosslinked PVA materials using a simple one-pot method that eliminates the need for additional antifreeze and conductive agents. This approach enables the preparation of conductive gels with enhanced toughness, swelling resistance, and freezing resistance, making them suitable for flexible sensor applications in harsh environments. By altering the acylation and covalent cross-linking of PVA, we enhance the mechanical robustness of the gel while regulating its resistance to swelling. The presence of salt ions generated from the reaction involving C_6_H_15_NHCl, along with the addition of a salt solution like KCl to cease the reaction, are crucial factors for enhancing the electrical conductivity. The crosslinked PVAC-30 exhibits ~ 250% increase in mechanical strength compared to the original gel, while retaining excellent stretchability. This modification also reduces the gel’s hydrophilicity, significantly improving its anti-swelling properties and allowing it to maintain its morphology even after 30 d of underwater immersion. Strong interactions between DMSO and water molecules within the gel system, combined with the presence of salt ions, greatly enhance the gel’s antifreeze performance, enabling the organohydrogel to retain flexibility and high conductivity after freezing at − 20 °C. When applied in flexible sensor devices, the crosslinked organohydrogel demonstrates strong stability under both low-temperature and high-humidity conditions, maintaining stable signal detection after 24 h of freezing or underwater immersion. This organohydrogel sensing system shows promising potential in bionic robotic arm applications, achieving consistent signal interactions. This straightforward crosslinking method for PVA modification offers a viable approach for developing durable, swelling- and freezing-resistant conductive gels, providing new opportunities for flexible sensor applications in low-temperature and humid environments.

## Results and discussions

### Preparation of cross-linked organohydrogels

**Fig. 1:**
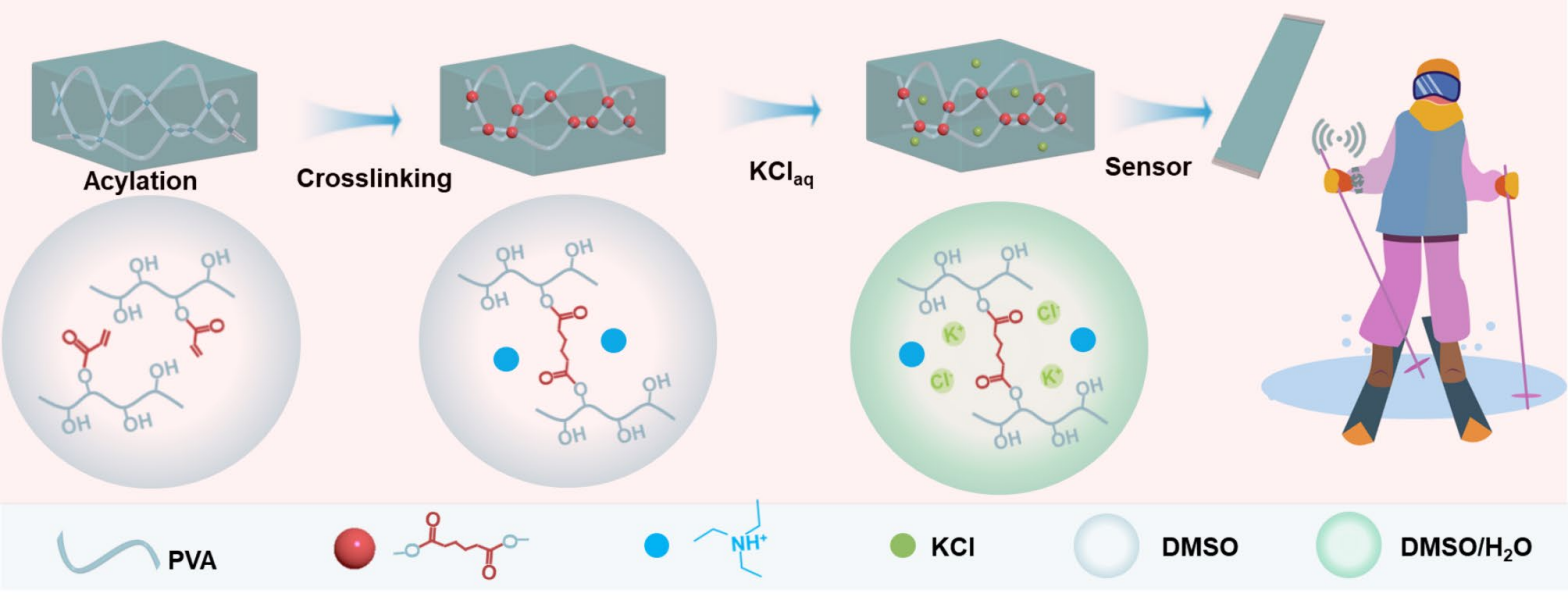
Preparation process and mechanism of organohydrogel.

To enhance the mechanical properties of PVA gels, we modified PVA chains with covalently crosslinkable double-bond, facilitating the formation of stable, swelling-resistant organohydrogel networks. As illustrated in Fig. 1, a conductive, flexible gel system with robust mechanical stability, anti-freezing, and anti-swelling properties was synthesized using a one-pot method, enabling reliable sensing performance under extreme cold and underwater conditions. During synthesis, some hydroxyl groups on the PVA chain reacted with acryloyl chloride (AC), grafting double bonds onto the macromolecular chains. Triethylamine (TEA), as the acid-binding agent, was added to neutralize the HCl produced, driving the acylation reaction forward. To mitigate potential toxicity and environmental impact, aqueous solution of KCl was added to neutralize residual AC forming acrylic acid and HCl as by-products. Crosslinking between the double bonds on the PVA chains was initiated by an initiator. By controlling the amount of AC, we could dynamically adjust the gel’s mechanical properties and swelling behavior. Furthermore, the reaction by-product, triethylamine hydrochloride C_6_H_15_NHCl, synergized with salt ions to enhance the gel’s conductivity and antifreeze characteristics, eliminating the need for additional conductivity or antifreeze agents to achieve multifunctional characteristics.

**Fig. 2:**
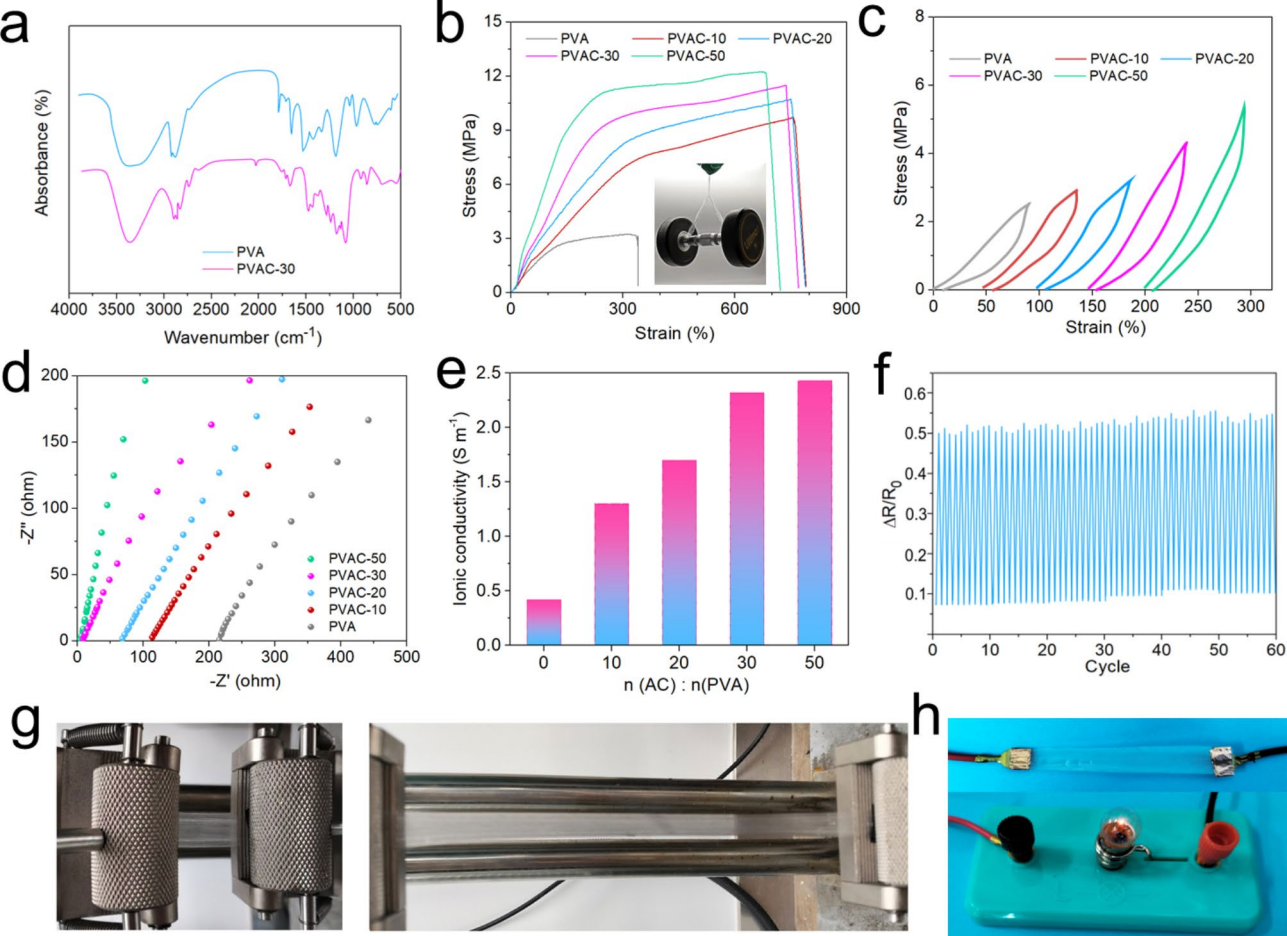
(**a**) Infrared spectra of PVA and PVAC-30. (**b**) Tensile strength test of organohydrogels, internal inset shows PVAC-30 gel easily lifting a 5 kg dumbbell. (**c**) Tensile loading-unloading test of gels (PVA, PVAC-10, PVAC-20, PVAC-30, PVAC-50) at a set strain of 100%. (**d**) Electrochemical impedance spectra and (**e**) ionic conductivity of organohydrogels. (**f**) Response and recovery times of organohydrogel sensors. (**g**) Tensile demonstration of organohydrogel PVAC-30. (**h**) PVAC-30 as a conductor of electricity to light a bulb.

To verify the successful synthesis of the material, IR and^1^H-NMR characterizations were performed. As shown in Fig. 2a, the –OH absorption peaks of the acryloyl chloride-modified PVAC-30 appear around 3400 cm^−1^. In contrast, the hydroxyl peaks of pure PVA are broad, indicating molecular aggregation, while the PVAC-30 shows weaker hydroxyl stretching peaks, suggesting partial consumption of hydroxyl groups during the reaction with AC. Additionally, the new absorption peaks at 1638.42 cm^−1^ and 800.57 cm^−1^ confirm the presence of C=C double bonds grafted onto the PVA molecular chain. The emergence of C–O–C peak at 1108.79 cm^−1^ further substantiates successful modification. The disappearance of double-bonded absorption peaks following thermal radical-initiated polymerization indicates completion of the cross-linking process. Moreover^1^, H-NMR spectroscopy (Fig. S1) supports successful synthesis of cross-linked materials. During acylation, the reaction generates HCl, which subsequently interacts with TEA to form C_6_H_15_NHCl, thereby enhancing the gel’s conductivity.

The mechanical strength of the organohydrogels was evaluated under dry state. The gels underwent a 12-hour desiccation at 60 °C to attain a dehydrated condition, expelling any remaining unbound DMSO and water, thus forming a compact gel void of free water content. In the strong hydrogen-bonding system of DMSO/water, organohydrogel PVA exhibits a tensile strength of 2.9 MPa and elongation up to 340%. Following cross-linking modification in PVAC-10, 20, 30, and 50, significant improvements in mechanical properties and flexibility were observed. As shown in Fig. 2b, the tensile strength of the PVAC-10 gel reached 10.2 MPa, with elongation up to 800%. This enhancement is attributed to covalent cross-linking, where lower cross-link densities increase the material’s flexibility, whereas higher cross-link densities induce rigidity, resulting in a brittle and less flexible structure^35^. As cross-linking density increases, this trend becomes evident: in PVAC-50, tensile strength rose to 12.0 MPa, though accompanied by a marked reduction in ductility. With an optimal crosslink density, PVAC-30 maintains high tensile strength along with substantial deformability. As shown in Fig. 2c, hysteresis curves under 100% strain reveal significant energy dissipation upon recovery. This behavior likely results from the irreversible breakage of certain covalent bonds or the reversible disruption of hydrogen bonds during stretching^37^. Compared to uncrosslinked organohydrogel, the crosslinked gel exhibits enhanced elasticity, attributed to its spatial network structure and stable covalent bonds, providing robust toughness under strain. This stability, supported by low-density crosslinking, positions the modified gel as a promising candidate for flexible sensing in applications that demand large deformations.

For flexible sensing applications, hydrogel materials should exhibit both excellent mechanical properties and electrical conductivity. Ionic conductivity in organohydrogels is typically assessed using electrochemical impedance spectroscopy. As shown in Fig. 2d, e, increasing the AC/PVA molar ratio from 0 to 50 enhances ionic conductivity. This enhancement is attributed to the simultaneous addition of AC, TEA (in a molar ratio equal to that of AC), and KCl solution during the terminated reaction. The increased addition of AC will lead to the formation of substantial amounts of C_6_H_15_NHCl. Within the mixed solvent environment of DMSO and water, enriched with salt ions, the hydrogel’s conductivity progressively increases from 0.39 S m^−1^ to 2.45 S m^−1^. This conductivity is partly attributed to the high dielectric constant of DMSO, which promotes ionic dissociation, and to the high solvent content within the hydrogel^38^. The DMSO contributes to a lower density of polymer chains per unit volume, thereby facilitating ionic transport within the hydrogel. Notably, without intentional conductive additives, the materials achieve conductivity solely through structural modification and cross-linking, which circumvents potential issues associated with the late-stage addition of conductive agents and antifreeze, such as inter-material incompatibility and reduced mechanical stability. Stable responsiveness is crucial for the effective application of sensor devices. As illustrated in Fig. 2f, the standard gel sample exhibits a consistent resistance change rate within a 100% deformation range. After 60 stretching cycles, this resistance change rate remains stable around 0.4, indicating excellent deformation responsiveness and stability. Figure 2g displays an image of PVAC-30 under 100% deformation, showing its flexibility and durability. As shown in Fig. 2h, the gel material is able to light up a bulb when integrated into a conductive circuit, demonstrating its conductivity under mechanical strain.

**Fig. 3:**
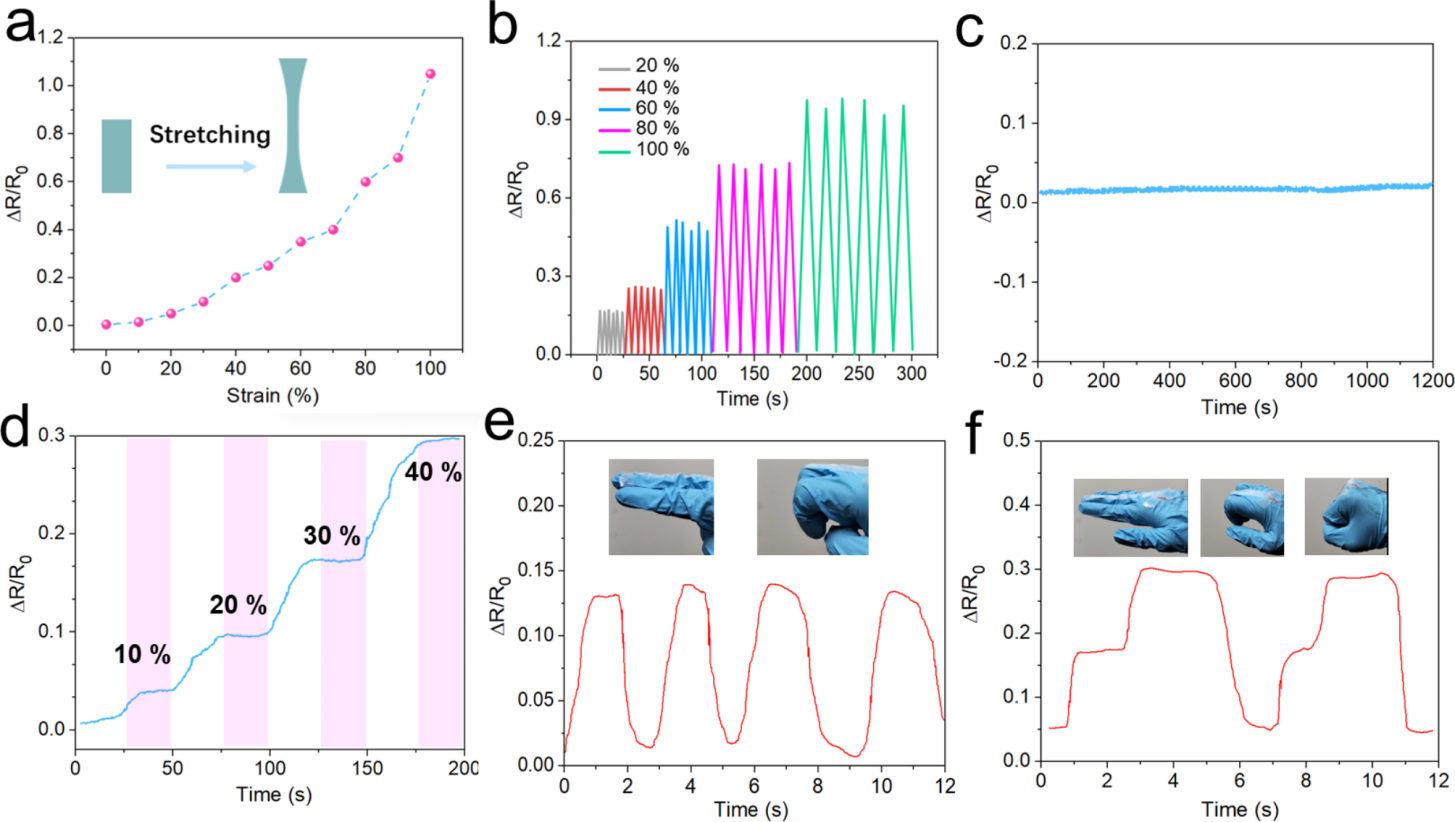
(**a**) Relative resistance change $\varDelta \text{R}/{\text{R}}_{0}$ of the gel strain sensor under different applied strains. (**b**) Resistance change curves of the gel under repeated cycles of 20%, 40%, 60%, 80% strain and 100% strain. (**c**) Long-term stability test of the gel sensor. (**d**) Stability test of relative resistance change at different strain levels. Application of organic hydrogel sensors for human activity detection: (**e**) finger bending and (**f**) fist clenching.

The organohydrogel functions as a sensor material, capable of detecting external deformation through changes in resistance, necessitating reliable deformation-resistance responsiveness and stability. As shown in Fig. 3a, the relative resistance change-strain response curve for the strain sensor shows that within a 100% deformation range, resistance change rate measurements increase incrementally at each 10% interval of stretching. This increase results from the progressive reduction in cross-sectional area under strain. The Gauge Factor (GF) of the flexible sensor was determined by analyzing the ratio between the resistance change rate $\varDelta \text{R}/{\text{R}}_{0}$and the strain ε exhibited by the organohydrogel. As shown in Eq. (1)^39^:

1 $$\text{GF}=\frac{\varDelta \text{R}/{\text{R}}_{0}}{{\upepsilon }}$$

The GF value of the prepared organic hydrogel reached 0.58 at 0–70% strain and 2.25 at 70–100% strain, and this sensitivity ensures the accuracy of this gel for the collection of limb activity signals. The sensor material maintains strain stability across additional tensile strain conditions, demonstrating responsiveness at 20%, 40%, 60%, and 80% strain levels (Fig. 3b). To further evaluate the stability of the electric skin strain sensor, resistance changes were recorded over approximately 1200 s, with minimal fluctuation observed throughout (Fig. 3c), confirming suitability for extended monitoring. Additionally, when the organohydrogel strain sensor was held at a fixed strain, the relative resistance change remained stable without significant drift, as shown in Fig. 3d, indicating consistent signal stability. Figure 3e, f illustrate the sensor’s application on a knuckle, where joint movement induces corresponding resistance changes, demonstrating high detection performance even for subtle motions like fist clenching.

**Fig. 4:**
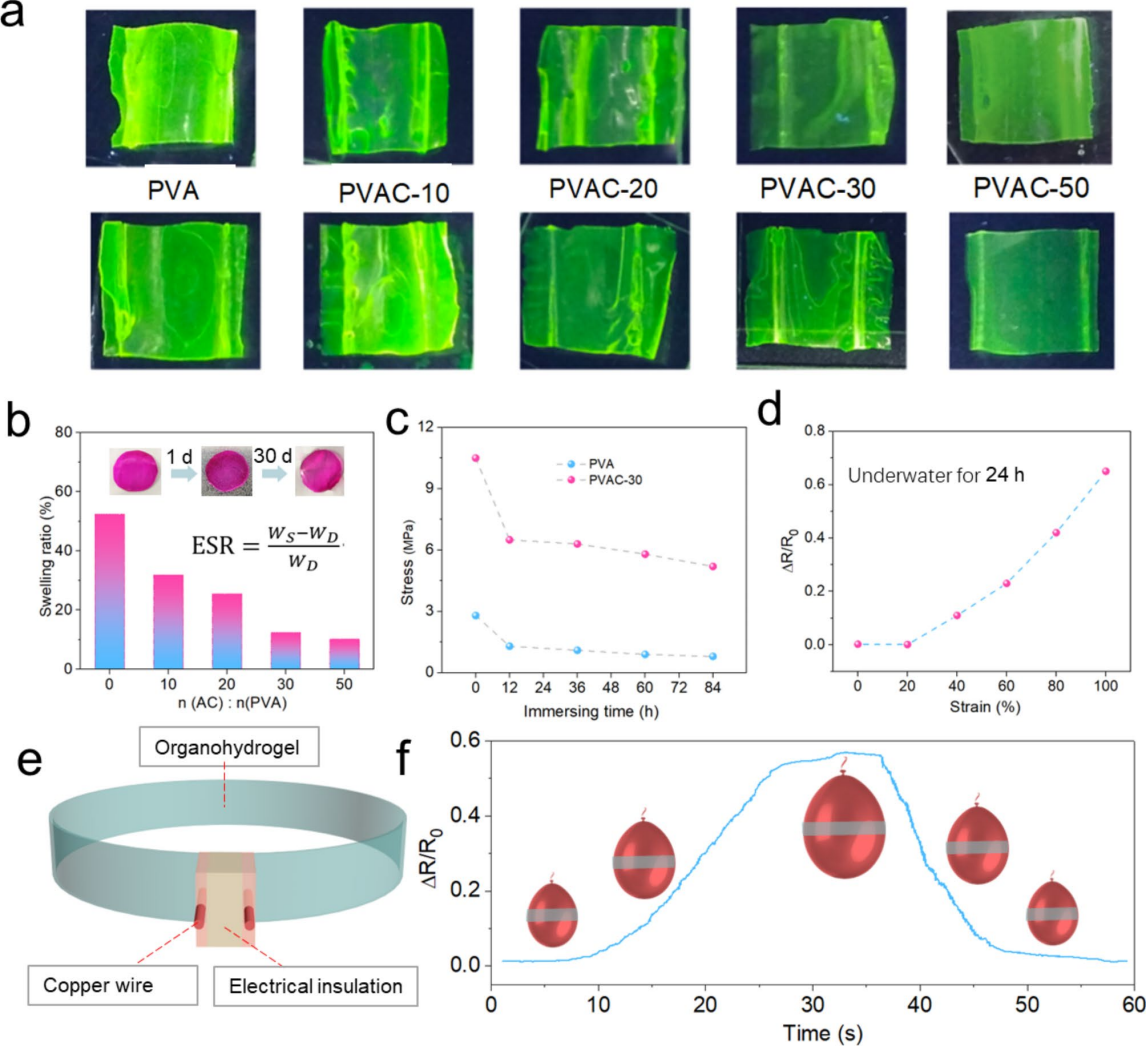
(**a**) Swelling tests of the gel in aqueous solution of fluorescent stain. (**b**) The swelling rate was calculated by swelling the gel in water for 24 h, and the inset shows the PVAC-30 gel before and after 30 d of swelling. (**c**) Tensile strength test of the gel after 24 h swelling. (**d**) Relative resistance change $\varDelta \text{R}/{\text{R}}_{0}$ of the gel after 24 h swelling at different tensile levels. (**e**) Conductive gel ring design. (**f**) Detection of balloon size underwater in a conductive gel ring.

Due to its hydrophilic nature, the PVA host material swells in aqueous environments, leading to significant decreases in mechanical strength and electrical conductivity, which may limit its effectiveness as a strain sensor. Modulating the cross-linking density along the PVA polymer chains allows adjustment of the gel’s anti-swelling properties. Water absorption in the prepared organohydrogels was assessed by immersing them in an aqueous solution with fluorescent stains for 24 h. After rinsing off surface stains, fluorescence imaging under UV light excitation showed the extent of fluorescent molecule penetration into the gel matrix (Fig. 4a). Notably, the PVAC-50 gel exhibited the lowest fluorescence intensity, indicating minimal penetration of fluorescent molecules despite its increased cross-linking density. This finding suggests that enhanced cross-linking decreases the gel’s solubility in water. To further quantify swelling efficiency, calculations were conducted using Eq. (2)^40,41^

2 $$\text{E}\text{S}\text{R}=\frac{{W}_{S}-{W}_{D}}{{W}_{D}}$$

${W}_{S}$ and ${W}_{D}$represent the weights of the swollen and dried gels, respectively. After 24 h of immersion, the gel reaches a swelling equilibrium, and its swelling rate does not increase significantly. As shown in Fig. 4b, when the molar ratio n(AC)/n(PVAC) is 30 and 50 (corresponding to PVAC-30 and PVAC-50 gels), the swelling rates are 11.5% and 9.8%, respectively, significantly lower than the 52.3% observed in uncrosslinked PVA. This difference arises from the lack of covalent cross-linking in unmodified PVA, which permits extensive water absorption within the gel. In contrast, modified PVA chains have a reduced hydrophilicity due to the consumption of hydrophilic hydroxyl groups during cross-linking, forming a spatial network that restricts molecular chain mobility in aqueous environments and limits water intrusion. The reduction in light transmittance observed in non-cross-linked gels results from excessive swelling, which increases coating thickness and causes light refraction within the coating (Fig. S2). To evaluate the mechanical stability of these organohydrogels underwater, PVA and PVAC-30 gels were immersed in water for 84 h, and tensile strength was measured periodically. As shown in Fig. 4c, tensile strength significantly declined after 12 h due to water molecules competing for hydrogen bonds within the gel, thereby weakening inter-chain interactions. Upon achieving the point of maximum water saturation, the PVAC-30 gel exhibits a tensile strength measuring 5.8 MPa. Nevertheless, even after 24 h of immersion, the gel maintained a stable resistance change rate of 0.68 under 100% tensile deformation (Fig. 4d), supporting its potential for underwater sensing applications. Circular sensors made of organic hydrogels, copper wires, and electrical insulation have been developed for underwater deformation detection (Fig. 4e). Further testing involved applying the ring-shaped gel sensor affixed to a submerged balloon to measure dimensional changes underwater. After 60 s of inflation and deflation, the sensor reliably detected deformation, demonstrating strong stability and responsiveness in underwater environments (Fig. 4f).

**Fig. 5:**
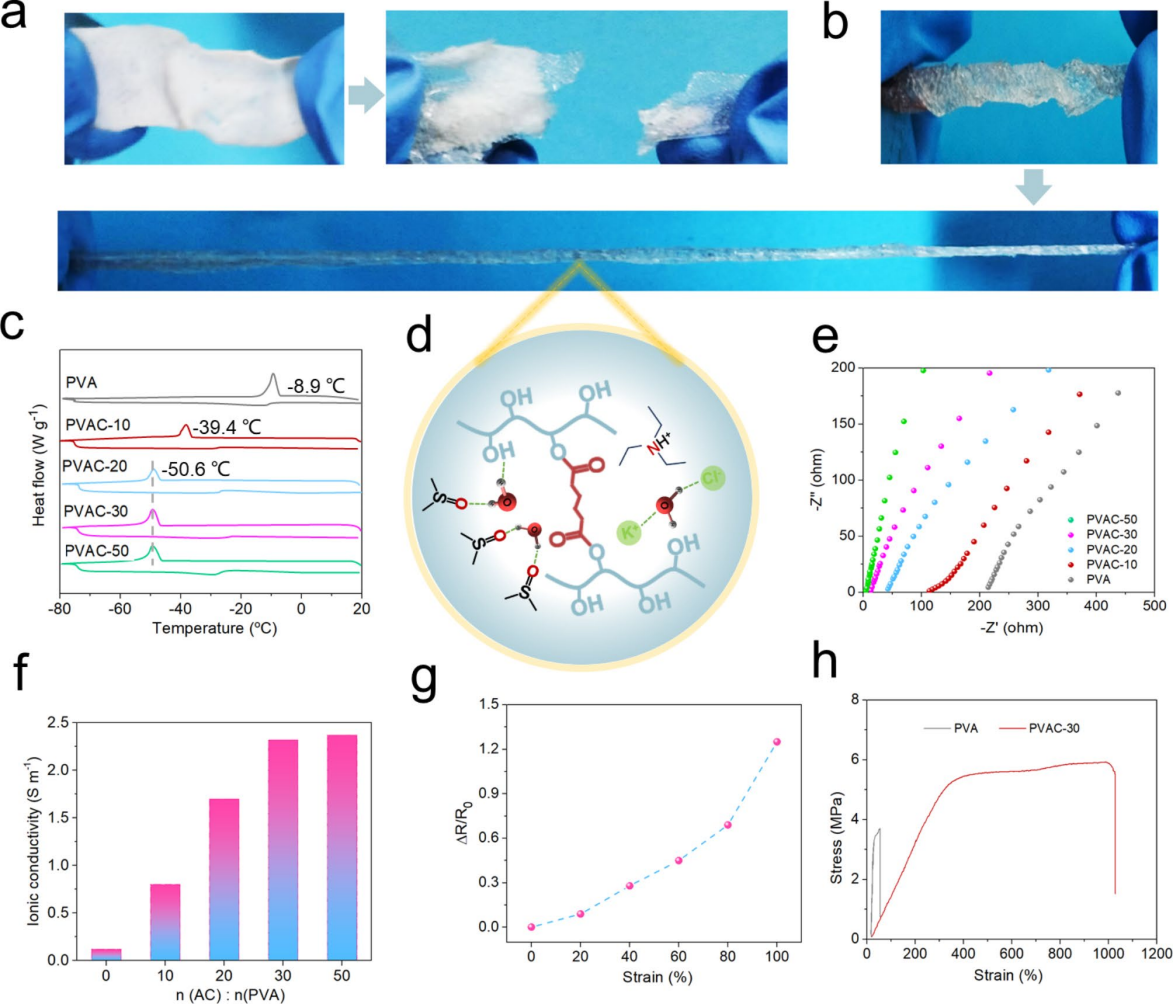
Anti-freezing effect as well as tensile demonstration of organogels after saturated with water absorption and frozen at -20 °C freezing layer for 24 h. (**a**) PVA, (**b**) PVAC. (**c**) Crystallisation temperature detection of different gels. (**d**) Anti-freezing mechanism of organohydrogels. (**e**) Impedance and (**f**) ionic conductivity detection of gels under freezing conditions. (**g**) Relative resistance change and (**h**) tensile strength detection of different gels under freezing conditions.

At low temperatures, water molecules within hydrogel networks form ice crystals through hydrogen bonding, causing a loss of elasticity and toughness, which limits hydrogel electrolyte applications in extreme conditions. To evaluate the anti-freezing performance of the gel material, prepared samples were stored at -20 °C for 24 h. After this period, PVA samples, which had absorbed significant water, were completely frozen, turned opaque, and fractured under tensile stress (Fig. 5a). In contrast, the cross-linked PVAC-30 gel retained greater flexibility under the same conditions (Fig. 5b). To further analyze the phase transition behavior of organohydrogels at low temperatures, freezing points were measured using differential scanning calorimetry (DSC). As shown in Fig. 5c, the PVA hydrogel exhibited a distinct exothermic peak at -8.9 °C during cooling. With increasing crosslinking density, the exothermic peaks of organohydrogels shifted progressively to lower temperatures, stabilizing at −50.6 °C for PVAC-20, PVAC-30, and PVAC-50. This progressive reduction in phase transition temperature is attributed to three main factors (Fig. 5d): (1) higher crosslinking density and increased electrolyte concentration within the gel reduce its water absorption, thereby lowering free water content; (2) DMSO within the gel acts as a hydrogen bond acceptor, enhancing interactions between water and polar groups; (3) dissolved internal salt ions inhibit hydrogen bonding network formation between water molecules, slowing freezing^42^. This effectively impedes water molecule crystallization, further reducing the gel’s crystallization temperature.

In addition to evaluating the phase transition temperature, electrochemical impedance spectroscopy (EIS) was used to measure the gels’ conductivity and further assess their anti-freezing properties. As shown in Fig. 5e, the gel’s impedance primarily ranges from 30 to 220 Ω. With increasing crosslink density, impedance decreases, indicating stable conductivity at low temperatures. Remarkably, the ionic conductivity of the PVAC-30 reaches 2.37 S/m at −20 °C (Fig. 5f). Under these low-temperature conditions, as depicted in Fig. 5g, the gel’s responsiveness was assessed by measuring resistivity changes over a 0–100% strain range. The relative resistance change of the gel sensor increased progressively with deformation, demonstrating high sensitivity across a broad operational range, suitable for extreme cold environments. Moreover, the PVAC-30 organohydrogel retains high flexibility under freezing conditions, with a tensile strength of 5.8 MPa and extensibility exceeding 1000% of its original length. In contrast, uncrosslinked gels saturated with water exhibit no effective frost resistance (Fig. 5h), underscoring the enhanced antifreezing properties achieved through crosslinking. If the covalently crosslinked network breaks, the organohydrogel exhibits self-repairing properties through hydrogen bonding, rebonding broken surfaces and restoring electrical conductivity (Fig. S3).

**Fig. 6:**
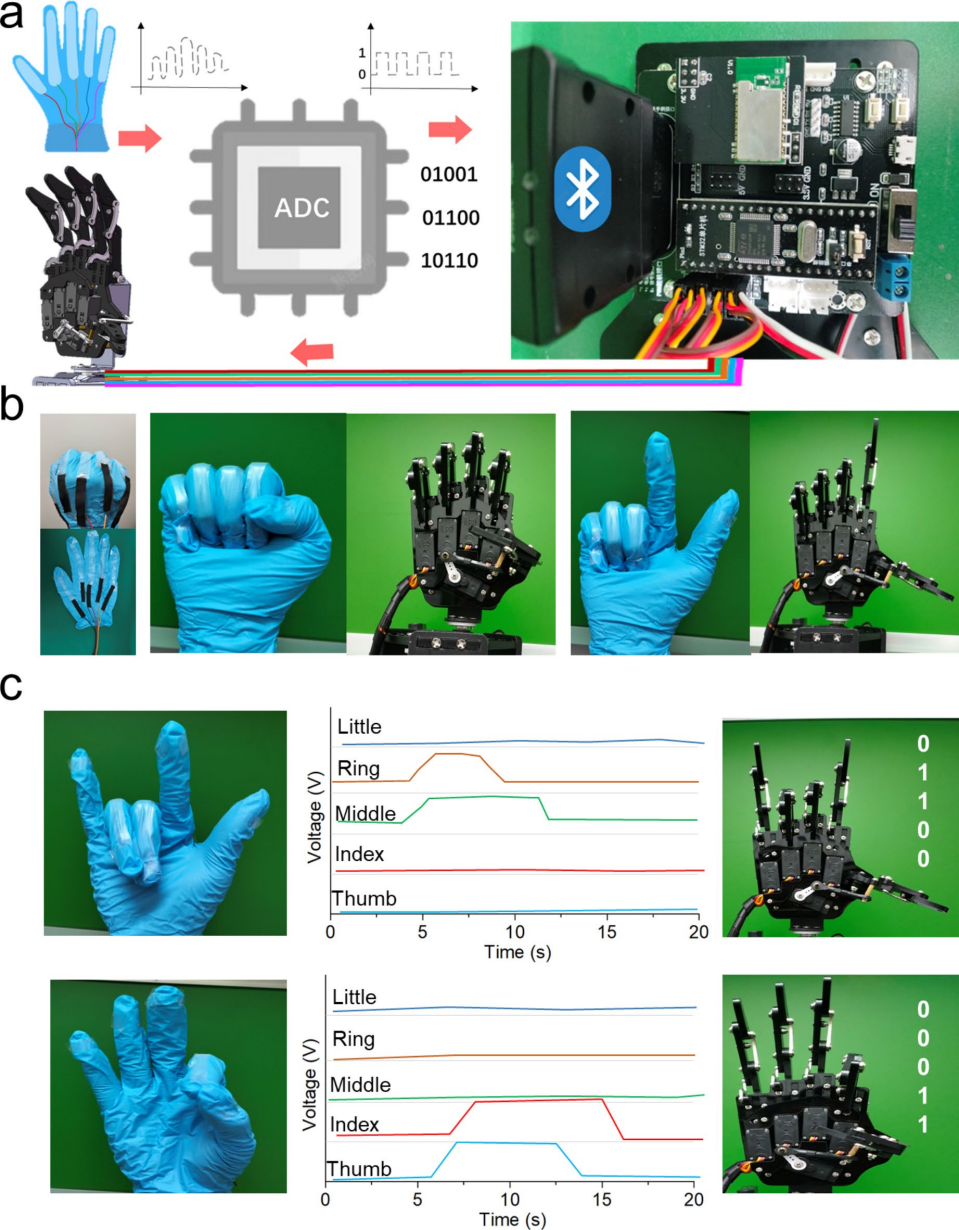
Glove-based intelligent control HMI system: (**a**) Flowchart of system operation. (**b**) Interaction of different gestures between human hand and robot. (**c**) Schematic diagram of normalised voltage signals in combination with strain sensor-based remote HMI.

Strain sensors are well-suited for monitoring hand joint movements, enabling the creation of a remote-control glove that integrates hydrogel sensors with wireless communication technology for long-range data transmission. Five hydrogel strain sensors were strategically positioned on the glove at each finger joint-thumb, index, middle, ring, and little finger-forming a sensor array. When a finger bends, the hydrogel channels compress, increasing resistance and subsequently raising the partial voltage across the trigger device.

The voltage is collected by a microchip, where an integrated analog-to-digital converter (ADC) converts it into a binary digital signal (0 or 1). The microcontroller then processes these signals and outputs control commands based on a robotic communication protocol, enabling real-time control of a robotic arm’s fingers through the glove (Fig. 6a). Figure 6b illustrates the glove’s remote control operation with the robotic arm, and Fig. 6c shows the schematic of normalized voltage signals for remote human-computer interaction. For example, performing an “I love you” gesture involves bending the middle and ring fingers along with the wrist, increasing voltage across the respective sensors while other fingers remain straight, resulting in a voltage signal coded as “001101”. Similarly, the “ok” gesture is represented by “00011”. The system demonstrates high accuracy in recognizing distinct gestures, proving adaptable to advanced human-computer interaction scenarios.

## Conclusions

This study synthesized a highly durable, electrically conductive hydrogel with anti-swelling and anti-freezing properties via a one-pot method, addressing limitations of conventional hydrogels in extreme environments. Through covalent cross-linking along the polyvinyl alcohol (PVA) chains, the gel achieves significant electrical conductivity and environmental stability without additional conductive agents or antifreeze components, while also exhibiting exceptional toughness. Experimental results reveal that the modified hydrogel has a tensile strength of up to 10.2 MPa, withstands over 800% elongation, and effectively responds to electrical signals in challenging conditions, including underwater and low-temperature environments. Its resistance to water and low temperatures underscores its potential for sensing applications in complex environments. Additionally, its integration into flexible sensors enables sensitive monitoring of limb movements with strong deformation resistance. Furthermore, when applied in remote control systems, the conductive hydrogel combines with wireless communication to enable precise robotic arm manipulation, suggesting promising applications in human-computer interaction and offering innovative pathways for advancing flexible electronics and bio-inspired smart devices.

## Materials and methods

### Materials

All chemical reagents were of analytical grade and used without further purification. Raw materials including poly(vinyl alcohol) (PVA-1799, hydrolyzed: 96–99%) and acryloyl chloride (AC, 97%), were purchased from Aladdin, and anhydrous dimethyl sulfoxide (DMSO, 99.7%), and triethylamine (TEA, 99.5%) were obtained from Beijing Chemical Reagents Co. Dibenzoyl peroxide (BPO, 99.0%), potassium chloride (KCl, 99.0%), The experimental water was deionised water.

### Acylation modification of PVA

5 g of PVA and the same molar amount of TEA as AC were weighed and dissolved in 100 mL of anhydrous DMSO and stirred at 60 °C until complete dissolution. The solution was cooled and the acylation reaction was carried out under ice bath conditions. Reagents were added at AC: PVA molar ratios of 0, 10, 20, 30, and 50. 0.026 g, 0.052 g, 0.078 g, and 0.13 g of AC were added to the PVA solution, and the AC was first dissolved in 10 mL of anhydrous DMSO and added drop by drop to the PVA solution. After 24 h of reaction, the reaction was terminated by adding 20 mL of KCl solution (2.0 M) to the gel solution.

### Preparation of crosslinked PVA gels

The acyl-modified PVA gel solution was added dropwise to a 2% mass concentration of 10 mL of BPO solution, and the reaction was carried out with rapid stirring under conditions after 75 °C, and the change in the viscosity of the solution was monitored during the reaction until a homogeneous and transparent gel solution was obtained. The gel solution prepared above was injected into a polytetrafluoroethylene mould (internal cavity volume 1 cm × 1 cm × 10 cm), vacuum degassed to an oven at 60 °C for 12 h, and the internal unbonded water and DMSO were removed to obtain a block gel. The above block gel was immersed in KCl solution (2.0 M, 100 mL) for 12 h to equilibrate. The samples were noted as PVA, PVAC-10, PVAC-20, PVAC-30, PVAC-50 based on AC to PVA molar ratio.

### Characterisation

A detailed characterisation section can be found in the supplementary information. Including specific characterisation methods, infrared spectroscopy, nuclear magnetic resonance NMR, differential scanning calorimetry DSC, mechanical properties of materials, electrochemical resistance, electrical conductivity, solubility, resistance change rate, material transmittance.

## Electronic supplementary material

Below is the link to the electronic supplementary material.

Supplementary Material 1

## Data Availability

Data is provided within the manuscript or supplementary information files.
