# Supplementary material for: Facile synthesis of ultratough conductive gels with swelling and freezing resistance for flexible sensor applications
Source: Sci Rep. 2025 Mar 1;15:7335. doi: 10.1038/s41598-025-86541-7 (PMC11873186; doi:10.1038/s41598-025-86541-7)
Supplement: Supplementary file 1 — Supplementary Material 1 [file 41598_2025_86541_MOESM1_ESM.docx]

**Facile Synthesis of Ultratough Conductive Gels with Swelling and Freezing Resistance for Flexible Sensor Applications**

***Pengpeng Lu^1, 2^, Jingyang Xu^3^, Shuyan Liu^3^, Lili Fu^3^, Shengxian Wu^1^, Ze Liu^1^, Tu Hou^2^, He Liu^1, 2*^, Dongyan Huang^1^***

^1^College of Engineering and Technology, Jilin Agricultural University, Changchun 130118, PR China

^2^College of Information Technology, Jilin Agricultural University, Changchun 130118, PR China

^3^Key Laboratory of Bionic Engineering, Ministry of Education, Jilin University, Changchun 130022, China

Corresponding author.

*E-mail addresses:* *liuhe@jlau.edu.cn*

**Characterization:**

Hnuclear magnetic resonance (1H-NMR) spectra were collected on a Bruker Advance III 400 MHz spectrometer with DMSO-*_d6_* as the solvent. Light transmission over 400-800 nm was collected on a UV-vis spectrophotometer (Shanghai Spectrum Win-SP5.0) during anti-swelling test. The chemical composition of the PVAC-30 organohydrogel was characterized by PerkinElmer spectrophotometer FT-IR with a wavenumber ranging from 4000 to 500 cm^-1^. Electrochemical impedance spectroscopy (EIS, CHI660E) tests were used to evaluate the impedance of organohydrogel sensors. The strain sensing performance was evaluated by measuring the electrical resistance change of organohydrogels upon deformation. The resistance of organohydrogels was recorded using a digital multimeter (Keithley, DMM6500). The anti-freezing property of the organohydrogel was studied using DSC. The prepared organohydrogels were cooled from 20 to -80 °C and then heated to 20 °C at a rate of-5 °C min^-1^.

**Mechanical Performance Tests.**

The tensile tests were performed using a universal material testing machine (CMT5105). The organohydrogels were stretched at 120 mm·min^-1^ at 25 °C, and -20 °C. The cyclic tensile tests were conducted for 5 loading-unloading cycles at 120 mm·min^-1^ and strain of 100% without intervals between consecutive cycles.

**Ionic Conductivity Tests.**

The ionic conductivity of organohydrogels was measured using an electrochemical workstation (CHI660E) at 25 °C and -50 °C with a frequency range from 105 to 10-1 Hz. The ionic conductivity of organohydrogels was determined according to the following equation: 𝜎 = L/RA^1^, 𝜎 represents ionic conductivity (S/m), L represents thickness (m), A represents the effective area of the gel electrolyte (m^2^), and R represents the intercept of the AC impedance spectrum on the real axis (Ω). The electrochemical analyses of gels were performed using a scan rate of 10 mVs^-1^.

**Anti-swelling Property Tests**

The prepared gel was dried in a polytetrafluoroethylene mould to prepare a circular gel, the mass of the dried gel was weighed and recorded as $W_{D}$, the mass of the water absorbing saturated gel was recorded after immersing the dried gel in water for 24 h and the mass of the water absorbing saturated gel was recorded as $W_{S}$, and the dissolution rate of the gel was calculated using equation $ESR=\frac{W_{S}-W_{D}}{W_{D}}$.

**Anti-freezing performance**

A differential thermal scanner (DSC, TA instrument Co., America) was tested from -80 to 20 °C with a cooling rate of 5 °C min^−1^ to characterize the anti-freezing property of organohydrogels. Wipe away the water on the surface of the water-absorbing saturated gels, and place the organohydrogels in a refrigerator at -20°C for 24 h to detect the tensile strength, ionic conductivity, the resistance change rate of the sensing.

**
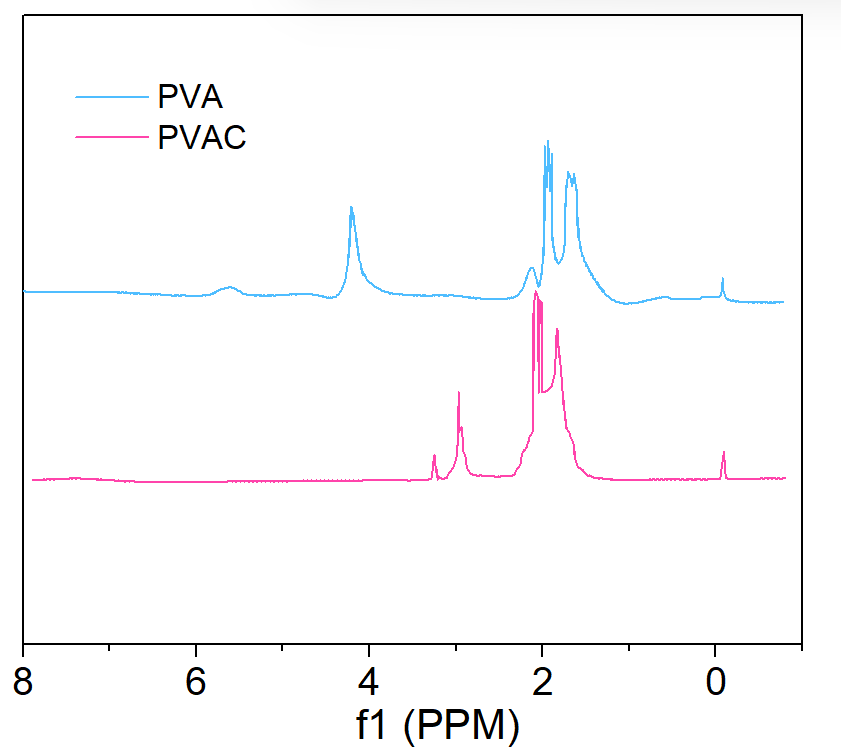
**

**Figure S1.** ^1^H-NMR spectra of PVA and PVAC

Due to cross-linking, the cured gel exhibited minimal solubility in deuterium reagents. ^1^H-NMR spectra, shown in Fig. S2b, reveal characteristic shifts: in unmodified PVA, δ = 1.6 (–CH– peak), δ = 2.095 (–CH₂–), and δ = 4.767 (–OH). In PVAC-30, peaks at δ = 1.810, 1.960, and 2.030 correspond to –CH–, with δ = 2.081 for =CH₂– and a CH₂=CHCO– peak near δ = 4.5. These IR and NMR spectra confirm the successful modification and cross-linking of PVA.

**
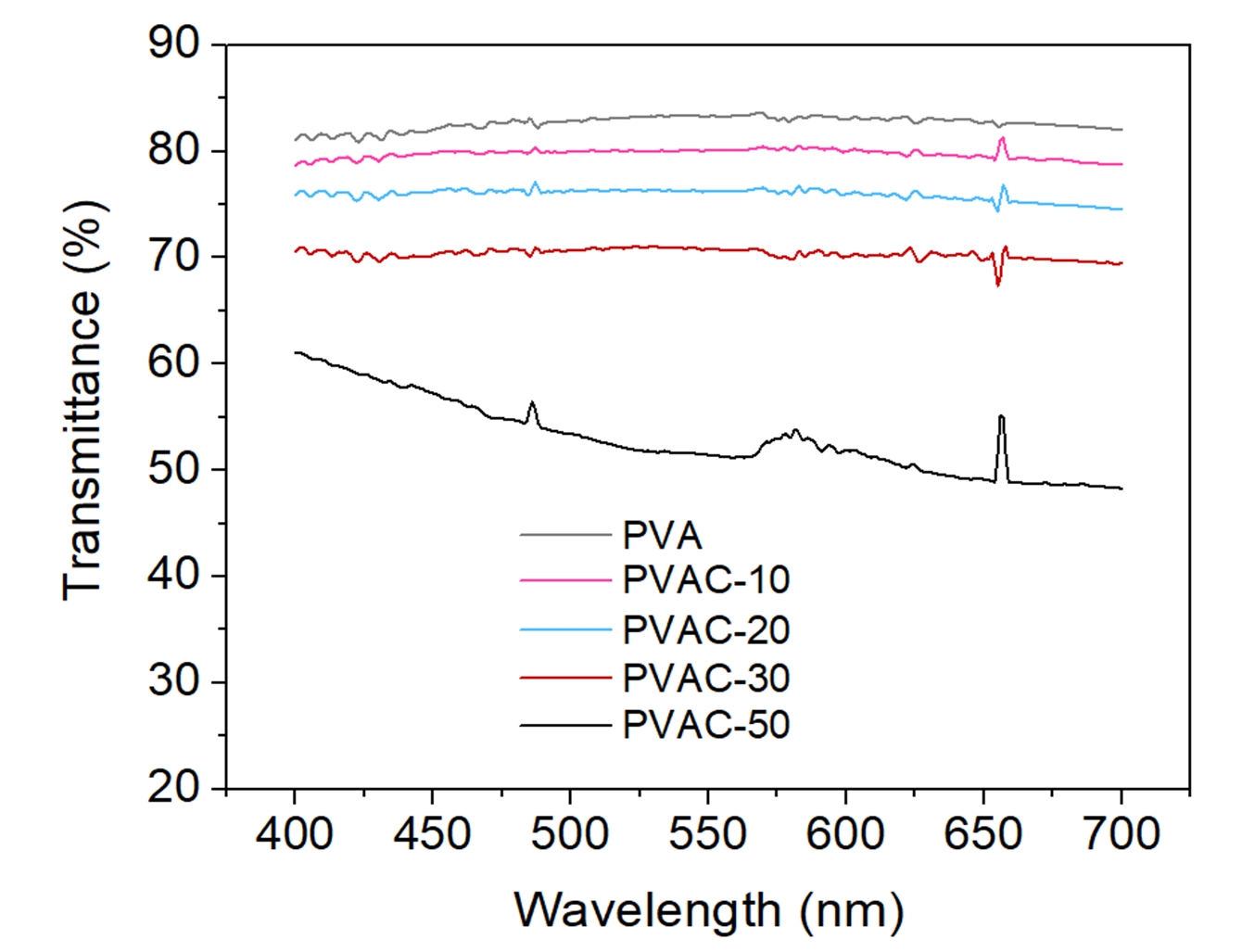
**

**Figure S2.** Transmittance of coating after 24h of swelling.

Transparent coatings of similar thickness were prepared by drop-coating the same volume of 150 μL of gel solution on the same area of glass substrate (25 mm×25 mm), and the thickness of the coatings was detected to be about 4.1 μm by using a varnish meter (DEMTGD31), and the transmittance of the coatings was detected by an ultraviolet spectrophotometer after 24 h of immersion. As shown in Figure S2, the light transmittance of the coating decreases with the decrease of crosslinking density, and the lowest light transmittance of the uncrosslinked organo-cement gel is only about 55 %, which is due to the excessive swelling of the coating surface and the refraction of the light inside the gel. The decrease in transmittance and the increase in refractive index also proved that the thickness of the uncrosslinked PVA gel coatings changed considerably^2,3^, which was caused by the large amount of water absorption and swelling.


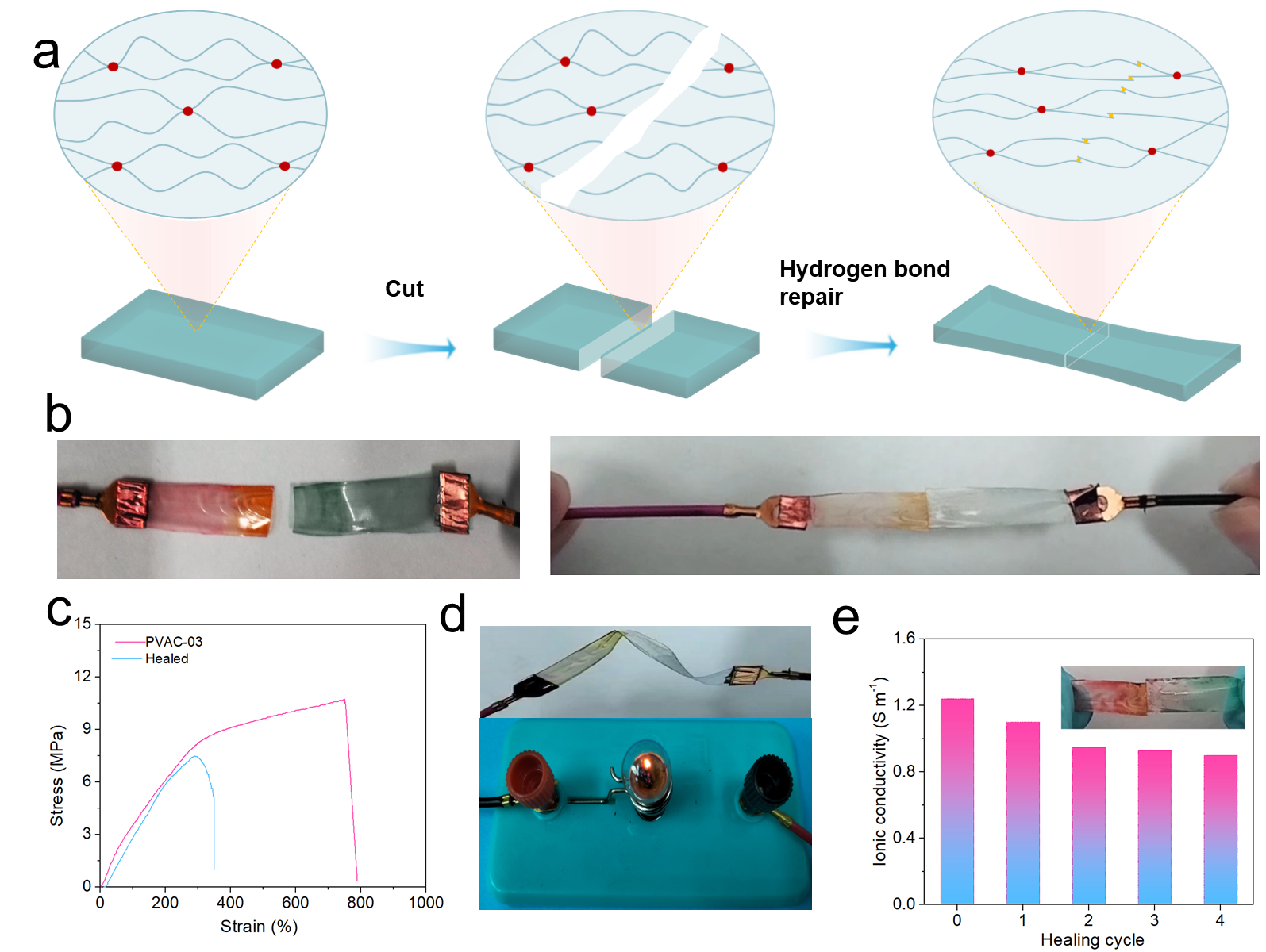


**Figure S3.** (a) Self-healing mechanism of organohydrogels. (b) Demonstration of self-healing effect of organohydrogels. (c) Tensile strength of the gel before and after repair. (d) Conductive lighting bulb of the repaired gel. (e) Changes in ionic conductivity of the gel during four repair cycles.

The gel structure, with its dual covalent and hydrogen bonding cross-linking, demonstrates excellent self-repairing capability through hydrogen bonding. Upon molecular chain breakage at the cross-section, numerous hydroxyl groups remain along the polymer chains, allowing hydrogen bonds at the interface to reorganize in the presence of the highly polar DMSO solvent, thereby re-establishing cross-linking (**Fig. S3a**). As shown in **Fig. S3b**, this self-repairing effect enables the gel sensor’s cross-sections to seamlessly reassemble without visible interfacial fracture under tensile stress. Following repair and drying, the gel sustains a stress of up to 7.5 MPa. Furthermore, the repaired gel retains sufficient conductivity to light a bulb (**Fig. S3c**). After four cycles of repair, the gel maintains an ionic conductivity of 0.9 S/m^-1^ (**Fig. S3d**), demonstrating robust and repeatable self-repairing performance.

**References**

1 Yu, J. *et al.* Highly Conductive and Mechanically Robust Cellulose Nanocomposite Hydrogels with Antifreezing and Antidehydration Performances for Flexible Humidity Sensors. *ACS Applied Materials & Interfaces* **14**, 10886-10897, doi:10.1021/acsami.2c00513 (2022).

2 Zhao, J., Meyer, A., Ma, L., Wang, X. & Ming, W. Terpolymer-based SIPN coating with excellent antifogging and frost-resisting properties. *RSC Advances* **5**, 102560-102566, doi:10.1039/c5ra21399a (2015).

3 Jena, K. C. & Hore, D. K. A simple transmission-based approach for determining the thickness of transparent films. *American Journal of Physics* **79**, 256-260, doi:10.1119/1.3533710 (2011).
